# Apelin elevates blood pressure in ICR mice with L-NAME-induced endothelial dysfunction

**DOI:** 10.3892/mmr.2013.1378

**Published:** 2013-03-15

**Authors:** KATSUMASA NAGANO, JUNJI ISHIDA, MADOKA UNNO, TANOMU MATSUKURA, AKIYOSHI FUKAMIZU

**Affiliations:** 1 Life Science Center, Tsukuba Advanced Research Alliance, University of Tsukuba, Tsukuba 305-8577, Japan; 2 Graduate School of Life and Environmental Sciences, University of Tsukuba, Tsukuba 305-8577, Japan

**Keywords:** apelin, APJ, L-NAME, endothelial dysfunction, impaired vasodilatation, mice, vasopressor action

## Abstract

Apelin is the endogenous ligand of APJ, which belongs to the family of G protein-coupled receptors. Apelin and APJ are highly expressed in various cardiovascular tissues, including the heart, kidney and vascular endothelial and smooth muscle cells. Although apelin exerts hypotensive effects via activation of endothelial nitric oxide synthase (eNOS), the ability of apelin to regulate blood pressure under pathological conditions is poorly understood. In the current study, N^G^-nitro-L-arginine methyl ester (L-NAME), a potent NOS inhibitor, was administered chronically, to induce peripheral vascular damage in mice. L-NAME-treated mice exhibited hypertension, increased vascular cell adhesion molecule-1 and plasminogen activator inhibitor-1 mRNA levels in the aorta and impaired vasodilatation associated with decreased aortic eNOS expression, consistent with endothelial damage. Three days following withdrawal of L-NAME treatment, the blood pressure response to apelin stimulation was assessed. Although apelin reduced blood pressure in non-treated mice, it was found to transiently elevate blood pressure in L-NAME-treated mice. These results indicate that apelin functions as a vasopressor peptide under pathological conditions, including vascular endothelial dysfunction in mice.

## Introduction

Apelin was originally identified in bovine stomach as the endogenous peptide ligand of the APJ receptor, which belongs to the G protein-coupled receptor family (1). Apelin and APJ are expressed in various cardiovascular tissues, including the heart, kidney and vascular endothelial and smooth muscle cells (2). Apelin has been shown to induce hypotensive effects via the activation of endothelial nitric oxide synthase (NOS) in vascular tissues (3). In addition, we previously revealed that APJ is involved in apelin-dependent hypotensive activity and the activation of endothelial NOS (eNOS) using APJ-deficient mice (4). Therefore, it has been hypothesized that apelin functions as a vasodilator under steady state conditions.

By contrast, it has also been reported that apelin contracts saphenous veins from which the endothelium has been physically removed *ex vivo*(5) and phosphorylates myosin light chain, the rate-limiting event for vascular constriction, in cultured vascular smooth muscle cells (6). Although apelin has been suggested to function as a vasopressor, there is no evidence indicating whether its vasoconstrictive properties are effective during blood pressure homeostasis.

In the current study, the apelin-dependent effects on blood pressure were analyzed during vascular endothelial dysfunction, the most common predisposing factor in various cardiovascular diseases, including hypertension, thrombus, stroke, renal failure and cardiac failure (7,8). To induce vascular endothelial damage in mice, N^G^-nitro-L-arginine methyl ester (L-NAME), a potent NOS inhibitor, was chronically administered (9–11). L-NAME-treated mice exhibited hypertension, increased expression of endothelial dysfunction-related genes and impaired vasodilatation. Under these conditions, apelin was revealed to transiently elevate blood pressure in L-NAME-treated mice, although a hypotensive effect was observed in non-treated mice. These results indicate that apelin has a pathophysiological role in mice with endothelial dysfunction.

## Materials and methods

### Generation of hypertensive conditions in mice

Animal experiments were performed using 2-month-old male ICR mice under conscious and unrestrained conditions. L-NAME (Sigma-Aldrich, St. Louis, MO, USA) was administered as previously described (12). Briefly, L-NAME was suspended in water (1 mg/ml) and the mice were allowed to drink freely for 1 month. The L-NAME-containing water was replaced every week. Following L-NAME treatment, the bottle was changed to normal water for 3 days to allow the L-NAME to be excreted from the mice. Age- and gender-matched control mice were used in all experiments. Animal experiments were approved by the Institutional Animal Experiment Committee of the University of Tsukuba. Experiments were performed in accordance with the Regulation of Animal Experiments of the University of Tsukuba and the Fundamental Guidelines for Proper Conduct of Animal Experiments and Related Activities in Academic Research Institutions under the jurisdiction of the Ministry of Education, Culture, Sports, Science and Technology, Japan.

### Aorta preparation and total RNA extraction

Aortas were excised from mice, the perivascular lipids were removed and the aortas were frozen rapidly in liquid nitrogen. Total RNA was isolated from the aortas using an RNAgents Total RNA Isolation System kit (Promega Corporation, Madison, WI, USA). Briefly, frozen aortas were ground to a powder using the Multi-Beads Shocker (Yasui Kikai Corporation, Osaka, Japan) suspended in denaturing solution and mixed thoroughly with phenol chloroform. The mixture was centrifuged (14,000 rpm at 4°C for 20 min) to collect the supernatant, following which Ethachinmate (Nippon Gene Co., Ltd., Tokyo, Japan) was added. Finally, total RNA was collected using the ethanol precipitation method.

### Gene expression analysis by real-time RT-PCR

Total RNA (~1 μg) was reverse-transcribed using a QuantiTect Reverse Transcription kit (Qiagen, Hilden, Germany) and then real-time quantitative PCR was performed using a Thermal Cycler Dice and SYBR Premix ExTaq II. Relative gene expression was determined by the ΔΔC_t_ method. The following primers were used for amplification: GAPDH, 5′-TGT GTC CGT CGT GGA TCT GA-3′ and 5′-TTG CTG TTG AAG TCG CAG GAG-3′; vascular cell adhesion molecule-1 (VCAM-1), 5′-TGA ACC CAA ACA GAG GCA GAG-3′ and 5′-GGT ATC CCA TCA CTT GAG CAG-3′; plasminogen activator inhibitor-1 (PAI-1), 5′-TCA GGA TCG AGG TAA ACG AG-3′ and 5′-TGA AGA GGA TTG TCT CTG CG-3′; Tie2, 5′-CTG AGA ACA ACA TAG GAT CAA GCA A-3′ and 5′-AAC AGC ACG GTG ATG CAA GTC-3′; eNOS, 5′-AAT TAA TGT GGC CGT GTT GCA-3′ and 5′-GCT CAT TTT CCA GGT GCT TCA-3′; CD31, 5′-AGG TGT GCG AAA TGC TCT CG-3′ and 5′-AAG GAA GAC TCT GAC TGC AAG-3′; CD31, 5′-AGG TGT GCG AAA TGC TCT CG-3′ and 5′-AAG GAA GAC TCT GAC TGC AAG-3′; APJ, 5′-CCT TCT AGG TGT GCC TGT CAT G-3′ and 5′-CAC TGG ATC TTG GTG CCA TTT-3′.

### Measurement of isometric tension

Mice were anesthetized with isoflurane and thoracic aorta rings were excised in ice-cold Krebs-Henseleit buffer (NaCl, 118; KCl, 4.7; CaCl_2_, 1.8; NaH_2_PO_4_, 1.8; MgSO_4_, 1.2; NaHCO_3_, 25; and glucose, 11.1). The rings were cut into 3-mm sections and mounted in Easy Magnus system (Kishimoto Medical Instruments, Kyoto, Japan). The mounted rings were allowed to equilibrate for 60 min under passive tension of 35 mN in Krebs-Henseleit buffer gassed with 95% O_2_/5% CO_2_ at 37°C. During the following hour, the resting tension was increased twice to 60 mM KCl and once to 80 mM to optimize constriction. The rings were pre-contracted with noradrenaline [L-(−)-norepinephrine-(+)-bitartrate; Calbiochem, La Jolla, CA, USA] to achieve 20–30% of the maximal tone. Vasodilation was measured at increasing concentrations of acetylcholine (Ach; Sigma-Aldrich). Following the addition of each concentration of Ach, the subsequent dose was not added until the baseline had restabilized.

### Blood pressure measurements and intraperitoneal injection of apelin

Systolic blood pressure was measured using a programmable sphygmomanometer (BP-200; Softron Co. Ltd., Tokyo, Japan) using the tail cuff method as previously described (13). Prior to measurement, a 2-day preliminary examination was performed and the mice were familiarized with the sphygmomanometer. Unanesthetized mice were introduced into a holder mounted on a thermostatically controlled warming plate and maintained at 37°C throughout the measurement. Apelin was intraperitoneally injected while the mice were conscious and unrestrained. [Pyr^1^]-apelin-13 (Peptide Institute, Inc., Osaka, Japan) was suspended in saline (Otsuka Pharmaceutical Co., Ltd., Tokyo, Japan). Following measurement of basal systolic blood pressure, [Pyr^1^]-apelin-13 was intraperitoneally injected at 296 μg/kg body weight. Systolic blood pressure was measured continuously for ~5 min and data were collected every 20 sec.

### Statistical analysis

Statistical comparison was performed using GraphPad Prism version 5 for Macintosh (GraphPad Software, San Diego, CA, USA). Student’s t-test or Mann-Whitney U tests were adapted for the evaluation of the significance of the differences between groups. P<0.05 was considered to indicate a statistically significant difference. Results are expressed as the mean ± SEM.

## Results

### Hypertension in L-NAME-treated mice

**Figure 1:**
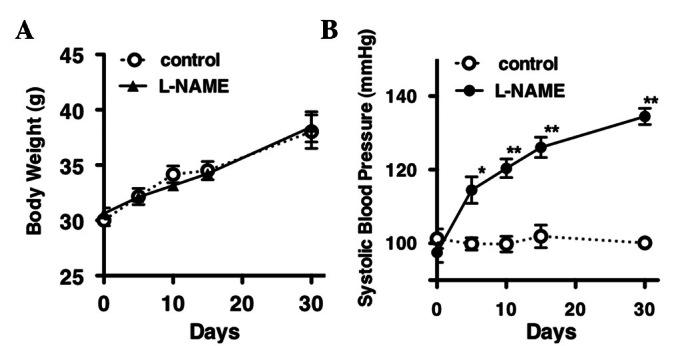
(A) Body weight and (B) systolic blood pressure of control and L-NAME-treated mice. Each parameter was measured at 0, 5, 10, 15 and 30 days following the start of treatment. Data represent the mean ± SEM. Each group, n=5–8. ^*^P<0.05 and ^**^P<0.01, vs. control. L-NAME, NG-nitro-L-arginine methyl ester.

L-NAME-induced hypertension in mice has been demonstrated to increase peripheral organ damage, including vascular endothelial dysfunction (14). To evaluate apelin-dependent blood pressure regulation during endothelial injury, vascular damage was induced in mice by L-NAME treatment. After administering L-NAME in the drinking water for 1 month, the body weight and blood pressure of non-treated and L-NAME-treated mice were measured. The body weight of the L-NAME-treated mice did not differ from that of the non-treated control mice (Fig. 1A). By contrast, the blood pressure of the L-NAME-treated group was found to significantly increase by ~20–30 mmHg (Fig. 1B, closed circles).

### Expression levels of vascular endothelial damage-related genes during L-NAME treatment

**Figure 2:**
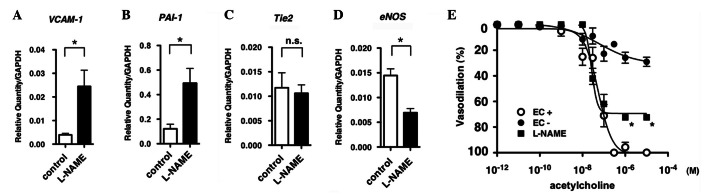
Evaluation of vascular endothelial dysfunction. (A–D) Analysis of gene expression of markers of vascular endothelial dysfunction. (A) VCAM-1, (B) PAI-1, (C) Tie2 and (D) eNOS mRNA levels were quantitated by real-time PCR. Each group, n=4–5 and ^*^P<0.05 vs. control mice. (E) Measurement of the vasodilative action of acetylcholine using an *ex vivo* assay in EC^+^ (control aorta), EC^−^ (aorta lacking endothelial cells) and L-NAME (L-NAME-treated aorta). Data represent mean ± SEM. Each group, n=3–5. ^*^P<0.01 vs. EC^+^. VCAM-1, vascular cell adhesion molecule-1; PAI-1, plasminogen activator inhibitor-1; eNOS, endothelial NO synthase; L-NAME, NG-nitro-L-arginine methyl ester; n.s., not significant.

To examine the effects of L-NAME on vascular injury, the expression levels of various endothelial damage markers were assessed, including VCAM-1 (15) and the fibrinolytic system regulation factor, PAI-1 (16,17). As demonstrated in Fig. 2, VCAM-1 and PAI-1 mRNA levels were found to be significantly increased in aortas obtained from the L-NAME-treated group, compared with those of the control (Fig. 2A and B). In addition, the gene expression levels of Tie2 and eNOS, a marker of vascular endothelial cells and an important vasodilative factor, respectively, were determined. Although Tie2 gene expression in L-NAME-treated and control aortas did not differ, eNOS gene expression was identified to be significantly decreased in the L-NAME-treated mice compared with the controls (Fig. 2C and D).

### Impaired vasodilation in L-NAME-treated aorta

Hypertension and vascular endothelial dysfunction are known to be major causes of impaired vasodilatation (15). Therefore, the acetylcholine-induced vasodilative activity of aortas obtained from L-NAME-treated mice was evaluated using an *ex vivo* assay. Aortic rings from the control group were completely dilated at a dose of 10^−6^ M acetylcholine (Fig. 2E, open circles). By contrast, aortic rings from which endothelial cells had been physically removed reacted minimally to acetylcholine (Fig. 2E, closed circles). For L-NAME-treated aortic rings, acetylcholine-induced vascular dilation was observed to occur in a dose-dependent manner, however, the maximum vasodilation was not reached completely (Fig. 2E, closed squares). Figs. 1 and 2 confirmed that endothelial cells remained structurally intact but were functionally damaged, as previously described (18).

### Hypertensive action of apelin in L-NAME-treated mice

**Figure 3:**
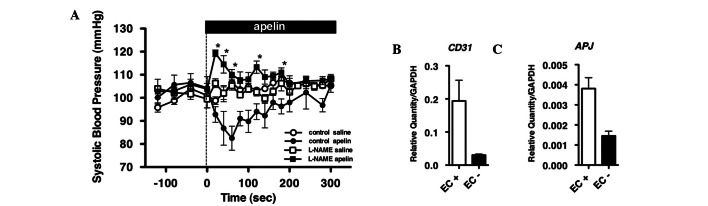
Vasopressor action of apelin in L-NAME-treated mice. (A) Time course of blood pressure alternation following intraperitoneal injection of [Pyr^1^]-apelin-13. Blood pressure at baseline was measured for ~100 sec prior to apelin administration. Following apelin injection, measurements continued for ~300 sec. Each group, n=3–4. ^*^P<0.05 vs. control apelin. (B) Remaining vascular endothelial cells were examined based on CD31 gene expression. Each group, n=3. (C) APJ gene expression in aorta tissues lacking endothelial cells. n=3–10. Data represent mean ± SEM. EC^+^, control aorta; EC^−^, aorta lacking endothelial cells; L-NAME, NG-nitro-L-arginine methyl ester.

To investigate the effects of apelin on blood pressure, apelin was administered to mice that were untreated or treated with L-NAME. In untreated mice, apelin administration transiently decreased blood pressure, compared with the effects of saline (Fig. 3A, filled circles). Of note, however, blood pressure was transiently elevated in the L-NAME-treated mice and the degree of increased blood pressure was significantly higher than that of saline-injected control mice (Fig. 3A, filled squares).

Finally, due to its importance in apelin-mediated hypertension, APJ expression in the aorta was examined using RT-PCR. Although the expression level of CD31, a marker of endothelial cells, was found to be significantly decreased by gently rubbing the aortic intimal surface (Fig. 3B), APJ gene expression was retained in the aorta (Fig. 3C). These results indicate that APJ is also expressed in smooth muscle cells, where it may regulate changes in apelin sensitivity following L-NAME treatment.

## Discussion

It is well known that systemic apelin administration releases vasodilatory substances, including NO, and lowers blood pressure (3,4). In the present study, the importance of apelin on blood pressure regulation under pathological conditions was analyzed by chronically treating mice with L-NAME (Fig. 1), an analog molecule of asymmetric di-methyl arginine (ADMA) that induces endothelial damage, one of the most serious factors in various cardiovascular diseases (7,8). L-NAME, like ADMA, inhibits NO production by suppressing the enzymatic activity of eNOS and induces vascular endothelial dysfunction accompanied by hypertension (14). Under these conditions, L-NAME-treated mice were confirmed to have hypertension, increased expression levels of endothelial dysfunction-related genes and impaired vasodilation (Figs. 1 and 2).

Treatment with L-NAME did not affect the expression levels of the Tie2 gene, a marker of vascular endothelial cells (Fig. 2C), indicating that endothelial cells were retained by the damaged vascular walls (Fig. 2A and B). By contrast, L-NAME treatment reduced eNOS mRNA levels (Fig. 2D). It has previously been reported that eNOS gene expression levels decrease upon L-NAME administration in rat aortic tissue (18). This observation is consistent with results of the present study and the reduced NO bioavailability and accelerated pathological conditions may be involved in the impaired vasodilation (Fig. 2E) associated with endothelial damage.

Under this pathological condition, apelin administration was found to provoke a vasopressor response, whereas it lowered the blood pressure of non-treated mice (Fig. 3A). The hypertensive action of L-NAME in mice may mediate the direct vasoconstrictive effects of apelin on vascular smooth muscle, since APJ expression was detected in aortic mouse tissues from which endothelial cells had been removed (Fig. 3B and C). Previously, it was reported that apelin passes through the ADMA-damaged endothelial barrier due to its increased permeability, as assessed by an *in vitro* assay (19). It is possible that apelin gains access to vascular smooth muscle cells during endothelial damage and constricts these cells.

In conclusion, the results of the present study indicate that apelin treatment in mice affects blood pressure in cases where blood pressure that is relatively low under normal conditions becomes elevated under the pathological conditions that are induced by endothelial dysfunction *in vivo*. Therefore, it is hypothesized that protection from endothelial damage is not only required to produce NO, but also to prevent the action of biological substances that constrict vessels. In this respect, the results of the current study provide a more detailed insight and an improved understanding of the complexities of blood pressure regulation by apelin.
